# Onco-Regulon: an integrated database and software suite for site specific targeting of transcription factors of cancer genes

**DOI:** 10.1093/database/baw116

**Published:** 2016-08-10

**Authors:** Navneet Tomar, Akhilesh Mishra, Nirotpal Mrinal, B. Jayaram

**Affiliations:** ^1^Supercomputing Facility for Bioinformatics & Computational Biology, Indian Institute of Technology-Delhi, New Delhi, India; ^2^Kusuma School of Biological Sciences, Indian Institute of Technology-Delhi, Delhi, India; ^3^Labaratory of Molecular Biology, South Asian University, New Delhi, India; ^4^Department of Chemistry, Indian Institute of Technology-Delhi, Delhi

## Abstract

Transcription factors (TFs) bind at multiple sites in the genome and regulate expression of many genes. Regulating TF binding in a gene specific manner remains a formidable challenge in drug discovery because the same binding motif may be present at multiple locations in the genome. Here, we present Onco-Regulon (http://www.scfbio-iitd.res.in/software/onco/NavSite/index.htm), an integrated database of regulatory motifs of cancer genes clubbed with Unique Sequence-Predictor (USP) a software suite that identifies unique sequences for each of these regulatory DNA motifs at the specified position in the genome. USP works by extending a given DNA motif, in 5′→3′, 3′ →5′ or both directions by adding one nucleotide at each step, and calculates the frequency of each extended motif in the genome by Frequency Counter programme. This step is iterated till the frequency of the extended motif becomes unity in the genome. Thus, for each given motif, we get three possible unique sequences. Closest Sequence Finder program predicts off-target drug binding in the genome. Inclusion of DNA-Protein structural information further makes Onco-Regulon a highly informative repository for gene specific drug development. We believe that Onco-Regulon will help researchers to design drugs which will bind to an exclusive site in the genome with no off-target effects, theoretically.

**Database URL:** http://www.scfbio-iitd.res.in/software/onco/NavSite/index.htm

## Introduction

DNA binding proteins routinely recognize their cognate sequences on genomic DNA in all living systems. While some of these interactions are sequence independent others are sequence specific, e.g. binding of a TF to its cognate motif is sequence dependent. As to how this sequence specificity is imparted remains a key question in biology. Crystal structures of different DNA motifs have revealed that local structure of DNA helix is sequence dependent (1–4). Various studies have highlighted that fine structural features of DNA such as helical twist, groove shape, slide, roll etc. to be sequence dependent (1, 5–7) which appear to be determinants for specificity in protein–DNA recognition. Several attempts are being made, to design molecules which can block DNA–protein interactions, the motivation being to design new antibiotics, antivirals and anticancer DNA-binding drugs (8). Many natural products like netropsin, distamycin, bleomycin, chromomycin, vinca alkaloids etc. are known to bind to DNA in a sequence specific manner (9, 10). While netropsin (11) and distamycin bind to AT-rich sequences, actinomycin D (12, 13) and echinomycin (14) bind to GC-rich sequences. Many of these molecules have therapeutic importance, e.g. Bleomycin is a glycopeptide antibiotic and induces break in the DNA after binding to it and hence it is used to kill cancer cells (15). These natural compounds usually have their molecular weight in the range of 500–2000 which can cover half a turn of the DNA helix. Their small size makes them attractive agents for treating cancer however it also makes them very non-specific and as a result these drugs have very high toxicity. The non-target binding and the resulting toxic effects of these small molecule drugs can be reduced by making their interactions with the cognate DNA specific and this remains a daunting task till date.

Clever concepts from synthetic chemistry are being used to design sequence specific DNA binding molecules for treating various diseases (16–18). However, little success has been achieved in designing synthetic molecules which can bind to DNA, sequence specifically at just one position in the genome (19–22). Many small molecules have been designed which sequence specifically bind to DNA but with off-target effects because their binding motifs are short and present at multiple locations in the genome. As a consequence, these drugs affect expression of many genes in addition to the gene of interest, e.g. p53 has around 400 target genes. Out of 400 if one gene gets mutated then better strategy would be block binding p53 in the promoter of that culprit gene without affecting its binding to 399 good genes. Hence, if we design a drug to inhibit p53 in order to control expression of the gene of interest then, theoretically, this drug will suppress the expression of other p53 target genes as well.

To overcome this problem, one needs to design a drug which binds at only one location in the genome. This requires the knowledge of TFs with a role in cancer initiation and or progression. Onco-Regulon is an effort in this direction. This is a database of > 200 TFs which have been implicated in different types of cancers like Carcinomas, lymphomas, Sarcomas, leukaemia and Adenoma. Information about transcription factors and their binding sites have been manually curated from either literature or databases. This database apart from providing complete information about different TFs implicated in cancer at a single platform also provides a way to uniquely target TF of choice by making use of the in-house developed programme unique sequence-predictor (USP) which predicts unique target motifs for these TFs in a gene specific manner. In our opinion, targeting of TF in gene specific manner can be done by targeting the DNA motif which is unique and is present only once in the genome, a task that is conceivable due to the availability of genomic sequences (NCBI). The core binding motifs of the TFs may be repeated many a times but the neighbouring sequences may not be repeated. Our hypothesis is to target the core DNA motif as anchor and the adjoining sequences for imparting singularity. An effective drug molecule would bind to not only the core of the cognate motif but also interact with the unique neighbouring sequences and hence would bind at only one location in the genome. Here, we present a web based tool that extends the core DNA binding motif at a given position in the neighbouring areas to predict unique sequence for each target motif of that particular TF. The core motif can be extended in 5′ → 3′, 3′ → 5′ and 5′← →3′ (i.e. all possible three directions). Thus, the software provides three unique sequences as output for each input sequence. Looking at the physico-chemical properties of the predicted sequences, the user can choose the best sequence for further analysis. As proof of the principle, we provide analysis of 51 breast cancer genes and provide unique targets for TFs regulating expression of these genes using USP tool. This can be done for other cancer types too. Accessing the database or the USP tool does not require any log in information and is available in public domain. Additionally, USP can be used in designing locus specific anchored primers for different repeat elements in the genome.

## Onco-Regulon database of TF binding sites

### Data collection and content

**Figure 1.**
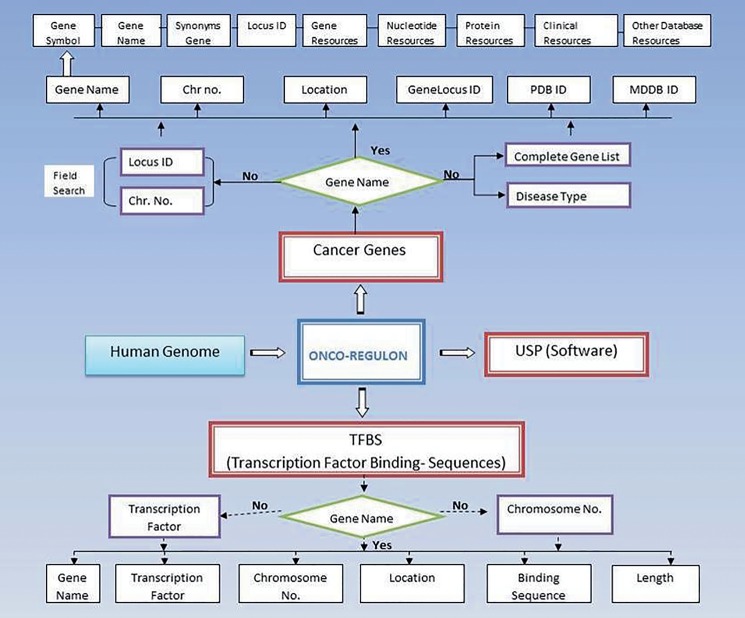
Schematic representation of Onco-Regulon. There are two main features of Onco-Regulon. One is the database part which comprises of a list of 933 genes implicated in cancer and another is the database of more than 10 000 transcription factor binding motifs in the human genome. Both databases are linked with human genome to provide position specific TF binding site information. The second main feature of the database is the software to predict unique sequence for sequence specific drug targeting and is named USP or Unique Sequence Predictor.

The Onco-Regulon is a collection of different transcription factor binding sites which play important role in cancer (Figure 1). The information about genes in different cancers was taken from Atlas of Genetics and Cytogenetics in Oncology and Hematology of National Cancer Institute. The information about TFs regulating these genes was taken from ChIP data available at SA Biosciences site which has been explained in detail below. Apart from this, we also performed literature search to catalogue functionally validated TF binding sites for all annotated genes implicated in different types of cancer. Further, this database in hyperlinked to various other databases and thus it acts as a super-database, one stop repository for cancer gene information.

**Figure 2.**
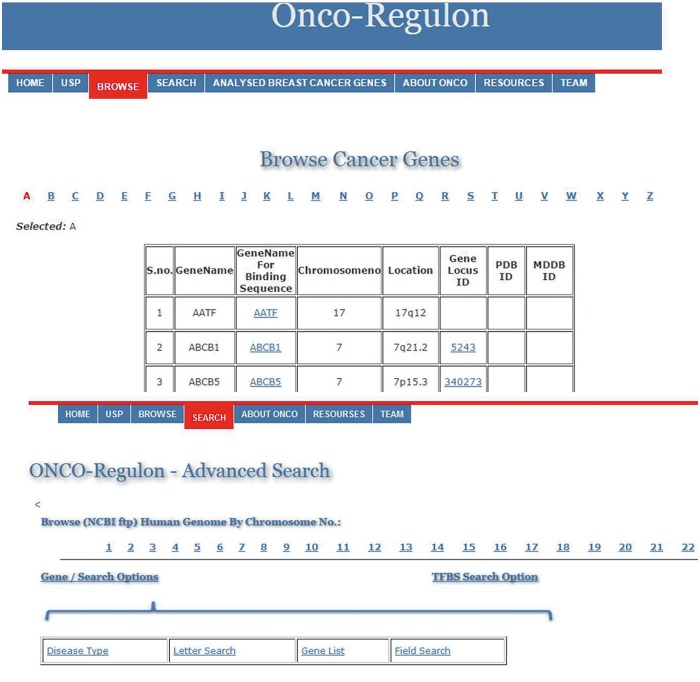
Using the database. The main database can be accessed by clicking the browse tab on the homepage. This returns a table of 933 cancer genes. The first column has gene name which is hyperlinked to provide basic information about the gene. The second column also has gene name but is hyperlinked to provide information about the transcription factors which regulate expression of the particular gene. This column is also hyperlinked and provides information about the TF binding sequence and position in the genome. This forms the basis for the position specific unique sequence prediction by the USP. Lower panel shows the advanced search option where the cancer gene database can be accessed by cancer type, gene name, field search or gene name letter search query.

(i) *Gene*. The first table contains the cancer genes under the tab named Cancer Gene. The last updated list has entry for 933 genes. The genes can be searched by first letter or by gene symbol under the Browse Cancer Genes tab. First column of the table has gene symbol which is hyperlinked to provide basic information about the gene name, location, locus id, chromosome no., gene product, gene resources (http://genome.cse.ucsc.edu; http://www.ensembl.org/Homo_sapiens/Gene; NCBI, EMBL, DDBJ), protein resources (http://www.uniprot.org/uniprot/), clinical information (http://www.omim.org), external database links (http://www.ncbi.nlm.nih.gov/CCDS/CcdsBrowse.cgi; http://www.ncbi.nlm.nih.gov/nuccore) and the last column mentions the references by pubmed (Figure 2). This basic information is present on the main page. After this all information about a particular gene entry is hyperlinked to various other databases. We also refer to some other useful databases like Go Pubmed (for literature mining to find target genes of a transcription factor), GeneCards, HGNC, Atlas of Genetics and Cytogenetics in Oncology and Hematology, NCI etc. to fetch a comprehensive list of genes involved in cancer. The genetic association of each gene with cancer was confirmed by OMIM database and expression analysis was confirmed by Uniprot. We have provided links to all these sites for cross validation by the users. So, users do not need to go to different sites for collecting different information. Oncoregulon provides link to all these tools along with the USP software on one platform. This enhances the ease of use for both cancer biologists and drug developers.

(ii) *Binding* *sequences* *database*. This is the most important aspect of the cancer gene database. The second column of the cancer gene table has the information about the regulatory motifs of all 933 cancer genes. This is accessed when the user clicks the Browse tab on the Onco-Regulon homepage. When the user clicks the gene name in the second column, a pop window opens with the details of the functional regulatory sites of the TFs which regulate expression of the gene in question. This provides information like name of the TFs, sequence of the binding motif along with the length and position in the human genome. So far, we have stored >10 000 binding sequences for all possible TFs of 933 cancer genes primarily from the ChIP data from the SA Biosciences website. For this, we considered all TFs that were found in ChIP analysis in the upstream 2 kb region of each of the 933 genes and the binding positions were manually curated in our database. Further, these binding positions were crosschecked in the UCSC genome browser by entering the gene name in the search window and analysing DNase Hypersensitive sites in the 2 kb upstream region. The cases where there was any conflict between the genomic locations of the TF in the gene, preference was given to the UCSC coordinates. Wherever possible we also looked for other experimental evidences like EMSA, reporter gene assay like luciferase and DNA protein structural complexes after searching the relevant literature. The search tracks for each gene can be found on the Go Pubmed link of individual gene page which opens after clicking the gene name from the gene list on main page. Users can also search the database using TF search option which allows the user to find binding sites of a given TF in 933 cancer genes. This not only returns gene list but also the co-ordinates of the binding site in the gene. We are continuously adding more information based on the new publications to make this database a huge one stop repository for sequence specific drug designing to cure cancer (Figure 2).

(iii) *Human* *genome*. In this section, we provide the human genome files for all 22 autosomes and X and Y sex chromosomes that are directly linked to the NCBI ftp genome directory. These gene files also support the search tools of USP to calculate frequency of any nucleotide sequence in complete human genome.

(iv) *Unique* *sequence* *predictor*. This in-house developed tool is part of Onco-Regulon, which predicts unique sequence for a given binding motif of TF of interest in target gene specific manner. Currently, this programme can only be used with human genome. The functioning of this programme has been explained in detail later in this manuscript.

After collecting the relevant information about genes, we combined all data in pocket by using the MySQL, RDBMS. This part of the database works in back end and the all web pages are connected to the database with the help of html and JSP.

### Implementation of unique sequence predictor

**Figure 3.**
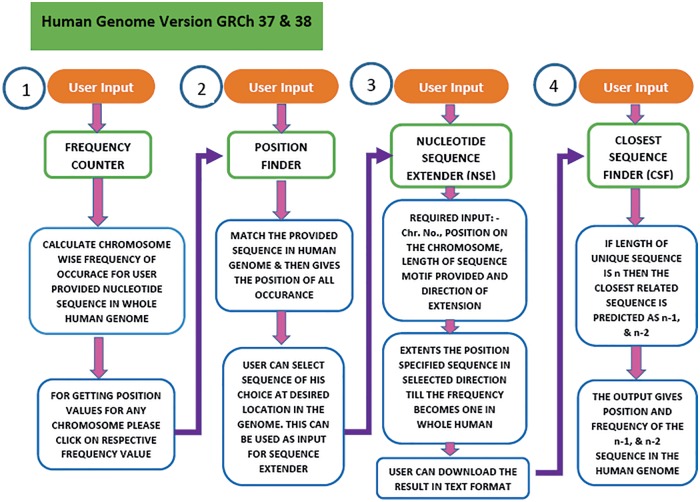
Workflow of the USP program. To predict the unique sequence for a given DNA binding motif the USP makes use of four different programs namely Frequency Counter, Position Finder, Nucleotide Sequence Extender and Closest Sequence Finder. All the four programs run in a stepwise manner but can also be used as independent tools. The same has been shown in this flowchart.

USP is written in *Perl* and PHP. The workflow in USP is composed of four sequential but integrated steps (Figure 3). The first step (Frequency counter) calculates the frequency of the sequence provided by the user in the entire genome. In second step (Position Finder), the position of the given sequence is searched in the genome. In the third step (Nucleotide Sequence Extender), the input sequence is extended in all possible three directions to generate three unique sequences for each input sequence. The fourth step is to find the sequence closest (*n* − 1, *n* − 2) to the unique motif (*n*), by the programme named Closest Sequence Finder. In the final step, we have provided links to four different softwares for further analysis of DNA groove, shape, interaction with molecules etc. under the heading Additional Analysis. Currently, the software is compatible with both GRCh37 and GRCh38 versions of the human genome.

### Input format for USP

The user can type or paste the DNA sequence of any length in the given window for each of the four program. No specific sequence file format is required.

### Frequency counter

Frequency counter is a tool that calculates total frequency of the given DNA sequence in the human genome (Figure 3). The user provides nucleotide sequence query in text field and upon submission gets the result in the same browser window. The results are presented in a tabular form where total occurrences of the initial input sequence are provided for both strands of each human chromosome. As per the requirement, the user can select the chromosomal strand to find position of sequence of choice. For higher sensitivity, specificity and accuracy, Frequency Counter programme works on exhaustive search algorithm with buffer variables to find exactly similar matches and does not allow any mismatch at any position. While the programme has been primarily developed to help design DNA binding drugs with little or no off-target binding, this can also be used for genomic analysis of any small sequence even a single nucleotide.

### Position finder

Many a times, the user may have a sequence but may not know the locus in the genome for that sequence. To design a locus specific drug one must to know the position of the drug binding site in the human genome. For fetching position of the input sequence we developed Position Finder. To use this programme, user can manually feed the sequence and select chromosome. Upon submission, user gets the starting position of the sequence on the selected chromosome. If the user starts from Frequency counter programme then he/she only needs to click the frequency result on the selected chromosomal strand and the sequence query gets autofilled in the query box of the Position Finder programme. The output is presented in a table and the user needs to select the position of interest. Selecting a specific position from this list gets autofilled in the query box of the Nucleotide Sequence Extender (NSE) programme. Thus, output of Position Finder becomes query for NSE.

### Nucleotide sequence extender

Nucleotide sequence extender (NSE) is the core component of the USP tool. Here, along with the sequence the user needs to define the co-ordinates of the given motif by providing information about chromosome number and strand, starting position of the motif along with its length. Knowledge of the exact position of the first nucleotide of the sequence motif is a must to fetch the right result. This information can be manually fed or selecting the position from the Position Finder output autofills all the information in the NSE query box.

**Figure 4.**
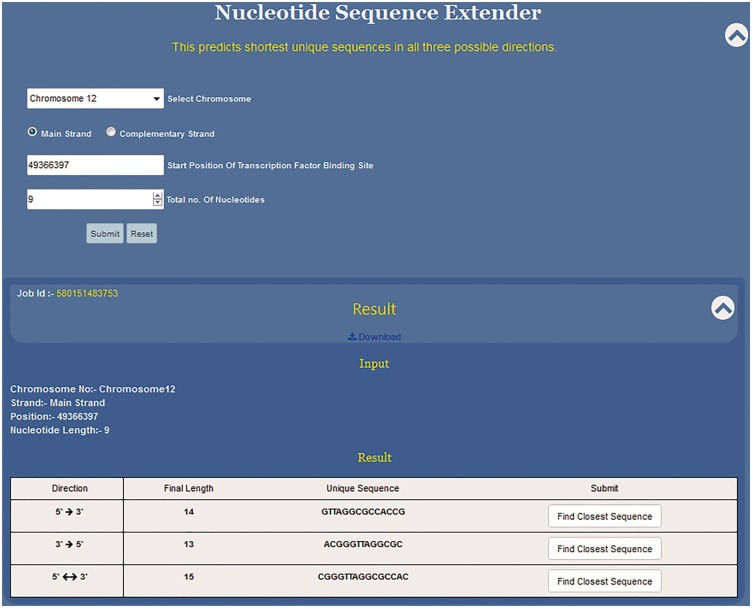
Functioning and output of nucleotide sequence extender. Nucleotide sequence extender program of USP requires information about chromosome no., starting position of the TF site. Output gives the unique sequence its length in all three directions so that the user can choose the best result. Result can be downloaded. Clicking radio button find closest sequence autofills the query fields in Closest Sequence Finder program.

Since the DNA sequence can be grown in any three directions, this programme has been divided into three subparts to extend the given sequence in 5′ → 3′, 3′ →5′ and 3′←→5′ directions (Figure 4). Specifically, the position of the first nucleotide of the sequence motif on the chosen chromosome is to be typed in the text window. In the third input box the user selects the total length of the input sequence motif (e.g. if the DNA binding motif is 10 bp long then 10 and if it is 6 bp long then 6). By providing these two pieces of information, the 5′ and the 3′ position of the sequence motif is defined. This is crucial as the NSE extender programme adds nucleotides to the 3′ end position of the given motif so that the sequence grows in 5′ →3′ direction and to the 5′ end for sequence extension in the 3′ →5′ direction. The programme adds one nucleotide at each step for unidirectional growth (5′ →3′ or 3′ →5′) and calculates the frequency in the genome of the extended sequence at each step (Figure 3). This process is iterated till the time the frequency of the extended sequence becomes unity in the genome. For the bidirectional sequence extension 5′&← → 3′ the strategy is a little different as this programme adds two nucleotides, one nucleotide at the 3′ end and another at the 5′ end at the same time. Thus, contrary to unidirectional growth algorithm that adds one nucleotide; bi-directional growth programme adds two nucleotides per iteration.

NSE is primarily meant to predict unique short sequences up to 30 nt long. However, if the input sequence is part of a long repeat then the programme cannot predict short unique sequences. Hence, we have split the NSE into two parts which work in succession. Initially for every input sequence the programme tries to find ≤30 nt long unique motif in all three possible directions, i.e. 5′ →3′, 3′ →5′ and 3′← → 5′.Thus, for each input sequence, the software can predict three unique sequence outputs, one output for each direction of sequence growth (Figure 4). All three results are displayed in a single tab which allows the user to compare the results and select the best result (Figure 4).

**Figure 5.**
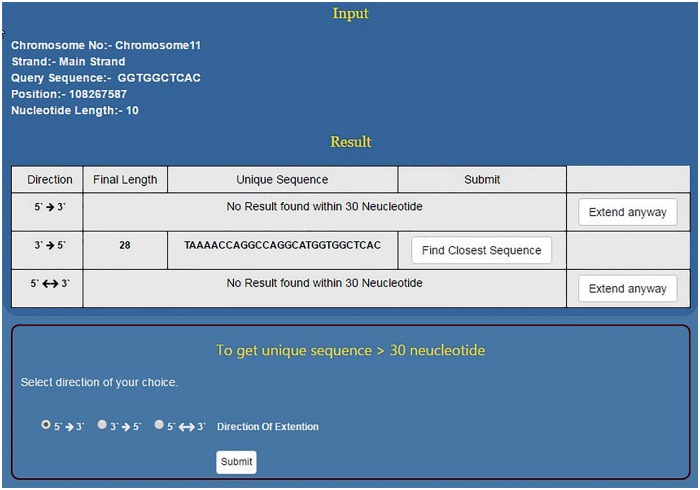
Using NSE for finding longer unique motifs. For a given short sequence, if NSE first module does not predict unique sequence up to 30 nt then the user has the option to go for second module which can predict unique sequence even few hundred nucleotides long. However, for the second module, user needs to select direction of sequence growth.

As mentioned above, NSE by default works to find a unique sequence which is ≤30 nt long. However, if the sequence doesn’t become unique within 30 nt then it flashes the message ‘No Result found within 30 Nucleotides’. At this stage, the programme prompts the user to use the second stage of NSE by clicking the button, ‘Extend Anyway’ (Figure 5). Once user selects this option he/she also needs to select the direction of growth and click the submit button. This programme will run till the sequence becomes unique in the genome or for 2 h, whichever is earlier. This will help the user to decide the priority and save time. If the priority is to get a short unique sequence the user will stop at module one. If the idea is to get a unique sequence of whatever length/genome analysis then the second module will be useful (Figure 5). In > 94% of the smaller motifs tested, USP returns ≤30 nt long unique sequence. However, motifs which do not become unique within this limit will be those that are part of long/short duplications in the genome.

### Closest sequence finder

Closest sequence finder (CSF) will find the frequency and position of closest sequence where the drug may bind non-specifically. The core logic is similar to Frequency Counter algorithm except that here whole human genome is searched for *n* − 1 and *n* − 2 motifs in one go. User can provide the input by selecting ‘Find Closest Sequence’ tab in NSE result window, which autofills required information for CSF. If the output sequence of the Nucleotide extender is *n* nucleotides long then this programme calculates frequency and position of the *n* − 1 and *n* − 2 nucleotides long sequences. To execute this program the pointer goes one or two steps back from the *n*th position and calculates the frequency of *n* − 1 and *n* − 2 nucleotide long DNA motifs. The idea behind this programme is to help the experimental biologist find the closest sequence for off target binding of the drug. Since *n* − 1 and *n* − 2 motifs will be the closest to the *n* nucleotides long unique motif hence these sites should be the most likely binding sites for the drug for the off-target effects. This is to be noted that the programme does not allow sequence mismatch as that will change the motif altogether. Only terminal length mismatches are allowed.

### Additional analysis

Once the user gets the unique sequence, naturally one would like to know the shape of the DNA corresponding to the sequence, major and minor groove dimensions, its ability to bind a small molecule etc. For all such analysis, we have provided four links for four different softwares (i) DNAshape, for major/minor groove analysis (23) (ii) DNA Sequence to Structure, (iii) DNA Ligand Docking, for docking small molecules (20) and (iv) PreDDICTA, for computing DNA–drug interaction energy (20, 21). All these programmes when used with USP can help to design a sequence specific drug on a single platform provided by Onco-Regulon web server.

### Output

As mentioned above, all four programs of USP are integrated in such a way that output of one program becomes input of the next program just at one click and user does not need to fill in the input every time. We have displayed on the right side panel the steps of USP as 1–5. One can key in the input in programme 1 and go to next step. This will reduce human error while using USP. At the same time, user has the freedom to use all the programmes independently. Once the unique sequence is predicted the user can download the result as .txt file. The result of NSE can be downloaded by its Job ID, just by selecting ‘Download’ button (Figure 4). For data security reasons, NSE results are encrypted and saved in SQL based database, so that if the user gives the same query again then the result appears within 2 s.

## USP: an application example

Onco-Regulon database has been developed to provide information for all cancer causing genes with an aim to design unique drugs which act in gene-specific manner without any off-target effects. Here, we show usefulness of the database using breast cancer as model cancer. *Step 1:* From the database we can fetch information for genes which are implicated in breast cancer by searching for genes by cancer type in the search column. A search for genes involved in breast cancer retrieves a table with a gene list of 312 genes. This list is not exclusive for breast cancer genes as same gene may be implicated in many different cancers. *Step 2:* From this list, we selected 47 genes based on literature survey and fetched DNA sequence of functionally validated regulators of these genes by clicking the Binding sequence column in the gene master list. The Onco-Regulon database predicted 275 regulatory sites in these 47 genes. See details in Supplementary Table S1.

*Step 3:* One of the genes frequently mutated in breast cancer patients is p53. Binding of p53 to the regulatory regions leads to transcriptional activation/suppression of the target genes. We looked at the Transcription factor column in the Table S1 and found out that out of 47 genes, 20 genes have p53 binding motifs. Few genes like DCC, NME, p53 and TGFα have multiple p53 binding motifs e.g. promoter of p53 gene has four functional p53 motifs. Thus, these 20 genes have a total of 27 p53 binding motifs (Table 1). Here, we have taken p53 as a model transcription factor and using the USP programme predict unique p53 binding sequences in the p53 target genes implicated in breast cancer. The p53 recognition motif is a decamer sequence to which the homo-tetrameric form of p53 protein binds (Table 1).

**Table 1.** Unique sequences identified by the Nucleotide Extender programme for 27 p53 Response elements from Supplementary Table S1

	Gene	p53 motif sequence	Frequency in the genome	Unique motif sequence (*n*)
1	ATM	AGACATGCTC	2552	AGACATGCTCAAGTTCT (17)
2	BCL2	ATCTGTACAG	3048	ATCTGTACAGACCTTAT (17)
3	BRCA1	TAGACATGTC	1852	TAGACATGTCTTTTCTTCCC (20)
4	BRCA2	AGGGATGCCC	15627	AGGGATGCCCTACCCC (16)
5	COL18A1	AGGCAGGCCC	23085	AGGCAGGCCCTCGGCA (16)
6	DCC-I	GAGCCTTCCTTGGCATTTC	2	GAGCCTTCCTTGGCATTTCA
7	DCC-II	AGACATGTCT	3868	AGACATGTCTTTGGCAC (17)
8	DCC-III	GAGCAAGTCCTGCCATGTT	2	GAGCAAGTCCTGCCATGTTA
9	ERBB2	AGCCATGCCT	4142	AGCCATGCCTGCGCA (15)
10	ESR1	GGTCATGCCT	2745	GGTCATGCCTGTAATCCCAGCACGTTGGGAGGCTGAGGT (39)
11	IGF1R	GGACACGCCC	599	GGACACGCCCCCCGA (15)
12	KIT	AGACATGGCC	2981	AGACATGGCCAATCAGC (17)
13	MDM2	CTGACTTGTCT	937	CTGACTTGTCTCCAGCTG (18)
14	MET	AGACATGCCT	4901	AGACATGCCTAATTTTTAT (19)
15	NF2	AGGCATGCGC	12392	AGGCATGCGCCATCCAT (17)
16	NME1-I	AGACTGGGCTGGGCATGGT	1	AGACTGGGCTGGGCATGGT
17	NME1-II	AGCCATGCCT	4142	AGCCATGCCTTTTCCCCAT (19)
18	NRAS	AGGGCATGCC	1980	AAAAAGAGAGGGCATGCC
19	PGR	GAGGCATTTC	3700	GAGGCATTTCTTCTATA (17)
20	PHB	TGGGGATGCC	3011	TGGGGATGCCCAGAGT (16)
21	TGFA-I	GAGACATGCC	2134	GAGACATGCCCACCTTG (17)
22	TGFA-II	CACCATGGCAGGGCCTTCC	1	CACCATGGCAGGGCCTTCC
23	p53-I	AGGGCAGGTCT	3855	AGGGCAGGTCTTGGCC (16)
24	p53-II	AGGGATGCCC	7687	AGGGATGCCCCAGAGCT (17)
25	p53-III	AGGCATGCACTACCATGCC	254	AGGCATGCACTACCATGCCCAGCTAATTTTTTTTTC (36)
26	p53-IV	GGACACGGACGGGCCTGGC	1	GGACACGGACGGGCCTGGC (19)
27	TSG101	AAGGCATGTA	3140	AAGGCATGTATCTAGG (16)

The numbers in the parentheses indicate the length of the extended unique motif. Second column shows the binding sequence on positive strand of DNA. Third column shows the frequency of the input p53 binding sequence. Last column shows the unique p53 target motif for each binding motif using nucleotide sequence extender program. Here, results are shown for the sequences grown in 5′ →3′ direction only.

*Step 4:* First, we use the Frequency Counter programme to calculate the frequency of each of these motifs in the human genome. From the table, it is evident that each of these motifs is repeated thousands of times in the genome. Cumulative frequency of all the listed p53 binding motifs is 78 732 (Table 1). This problem is further compounded by the repetitions of these motifs. Our analysis has shown that AGACATGCCT motif of MET gene is repeated 4901 times while the AGCCATGCCT motif present in the ERBB2 gene is repeated 4142 times (Supplementary Figure S2). Thus, the drug designed to specifically bind to AGCCATGCCT motif in the ERBB2 gene will likely bind at 4142 positions in the genome which means that it will have 4141 undesired bindings (Table 1 and Supplementary Figure S3).

*Step 5:* Next we used the Nucleotide Extender programme to predict unique sequence for each of these 27 p53REs. While the programme can extend each of these sequences in the three possible directions, here we have shown the results of sequence extension in 5′ → 3′ direction only (Table 1). The minimum length at which the p53 RE becomes unique is 13 for E2F transcription factor of WNT10B gene. Technically drug designing is more feasible for a shorter target then for a longer one. Thus, targeting a 13 bp unique motif may be more feasible. It is interesting to note that the 10 bp long p53 RE motifs of ERB-B2 and Met are 90% similar, with the sole sequence difference at the 3rd position (Table 1). This intuitively indicates that a drug designed to bind to p53 site in the ERB-B2 promoter may also bind to Met promoter. This will lead to off-target effect and may compromise the treatment. However, if the drug is designed to target the extended 15 bp long p53 unique sequence in the ERB-B2 promoter then cryptic binding of the drug at the Met promoter can be potentially avoided. Another problem foreseen here is that of sequence polymorphism. While our software works on reference genome the user is advised to take care of the polymorphism issue if any.

For further testing, the usefulness of the USP, we calculated the Tm of these sites using the software Tm-predictor (24). While the *Tm* of the 10 bp long p53RE of ERB-B2 and MET are 59.7 °C and 55.3 °C respectively (Supplementary Table S4). However, Tm of the 15 bp long extended p53RE of ERB-B2 is 70.4 °C (Supplementary Table S4). Thus, a drug designed to bind this to 15 bp long extended motif of ERB-B2 will be energetically not compatible with the 10 bp long p53RE of MET promoter. This shows that extending the DNA not only increases the sequence specificity but also the energy barrier between the target and the non-target sequence. Similarly, unique DNA targets can be fetched and analysed for other TFs or other genes. Thus, USP can be a great help in not only predicting but also physico-chemical analysis of the unique sequence for better drug designing.

### Analysing off-target sites of p53 in the genome

Since, the Nucleotide Extender programme stops at the first base (*n*) at which the frequency of the extended sequence becomes unity in the genome which, indicates that sequence of length (*n* − 1*)* will have multiple (more than one) binding sites in the genome. Thus, a drug designed to bind to motif of length *n* may cryptically bind to *n* − 1. Hence to test the efficacy and specificity of the molecule designed to bind motif *n* should also be tested for its ability to bind *n* − 1 motif as a control measure. We realized the importance of this information and hence developed the Closest Sequence Finder programme which predicts the position and frequency of the *n* − 1 and *n* − 2 motifs in the genome. We also calculated Tm for *n* − 1 and *n* − 2 motifs and compared with the Tm of the unique motif *n* for all the 27 p53REs. We found that if the last two nucleotides are G/C then the Tm difference between *n* and *n* − 1 and *n* − 2 motifs is significant (Supplementary Table S4). However, if the sequence becomes unique due to addition of A/T as the last base then the Tm of *n*, *n* − 1 and *n* − 2 motifs is almost similar (Supplementary Table S4). This suggests that the sequence which becomes unique due to addition of A/T may not be very attractive targets for drug designing from energetics point of view.

However, this sequence difference alone may be sufficient to impart a significantly different structure to the recognition motif. Now it is accepted that TF binding to the DNA depends not only on the sequence read out but also the shape read out. Hence, one needs to analyse the structure of the given *n, n* − 1 and *n* − 2 motifs for the difference in the shape readout to design a position specific DNA binding molecule.

To emphasize this point we analysed the major and minor groove geometry of the p53REs for which crystal structures are available. Since, in our analysis, we found that most of the sequence motifs become unique after reaching 20 bp sequence length hence we only selected those p53–DNA complexes where the DNA binding motif was 20 bp long. We found seven such p53–DNA complexes. The DNA geometry of these crystal structures was analysed using 3-DNA programme (25). From the minor and major groove width analysis of these seven structures, it is evident that each p53RE has a unique geometry and is different from each other (Supplementary Figure S5). This provides further support to our hypothesis that structural readout may be a stronger parameter for drug designing. This software USP is an attempt towards this goal.

## Discussion

Gene regulation is a complex process that is controlled by interactions of transcription factors (TFs) and co-regulators with the transcription machinery in the cell. Recent spurt in the availability of the ChIP and ChIP-Seq data has generated huge information about the occupancy of different TFs on a genomic scale (26). Analyses of these data have revealed (i) diversity in the binding motif sequence for many of these TFs and (ii) binding of many of the TFs in regions far away from genes. Typically, gene expression studies focus on regulators close to the transcription start site and hence, regulatory motifs which are far away from the TSS are often overlooked. This is due to (i) limitations of experimental approaches and (ii) many of the TF binding motifs are repeated multiple times in the genome. Most of the TFs have a few hundred binding positions in the genome, so how to account for the functionality of each of those binding positions is tough to answer.

Here, we have highlighted this point by analysing 21genes implicated in breast cancer which are regulated by p53. The p53 binding motifs in these 21 genes are different from each other which underlie the diversity in the p53 recognition motifs in the human genome. For example, the 10 bp long AG**A**CATGCCT motif present in the promoter of MET gene is closely related to AG**C**CATGCCT motif which is present in the promoter of ERBB2 gene. These two motifs differ in their nucleotide sequences at the third position. As a result, a drug designed to target p53 binding in the ATM gene may potentially affect p53 binding in the ERBB2 gene as well. Clearly any drug which is designed to target these decameric motifs will potentially bind at all those thousands of sites in the genome. This will affect the treatment in two ways: (i) the drug will affect p53 binding at multiple unwanted positions/genes apart from the target sites/genes and (ii) binding of the molecule at thousands of sites will potentially titrate the effective concentration of the drug required at the targeted site. This will have negative impact on the treatment regime. This problem of off-target binding can be overcome by designing drugs which bind only at the p53RE in the target gene. Since the drug has to bind in a sequence specific manner hence the target sequence has to be unique.

To unravel the functional significance of these DNA–protein interactions at each position one needs to develop tools which abrogate these interactions not only in sequence specific but also in a position specific manner. Traditionally genetic approaches have been used to answer these questions however it is not feasible to analyse all these interactions using genetic approaches because of the large volume of the data. A recent study identified 65 572 p53 specific ChIP fragments in the human genome which suggests that p53 protein physically interacts with human genome at 65 572 locations *in vivo*. Effect of p53 binding at the 65 572 locations can be studied by uniquely targeting these locations such that p53 binding at rest of the 65 571 locations is not affected. In our view, this can be done by identifying unique sequence around the core p53 binding motif. Since Frequency Counter programme allows the researcher to predict the unique sequences at the desired location in the genome along with the chromosome-wise distribution hence the researcher would be better able to design a strategy or a drug to study the regulation of gene on interest. Currently available frequency counter programmes like FIMO return frequency of the input sequence as output while USP predicts frequency of each extended sequence as output till the unique sequence is found in the genome (27). Further, USP also predicts closest sequence along with their locus in the genome and thus user has a better control over the information for designing experiments.

Recent studies in Drosophila have suggested that specificity in target recognition by Hox proteins is determined by minor groove shape while that of the Dorsal target genes is ascertained by the major groove geometry (3, 28, 29). This has brought focus on the shape readout of the DNA motif in determining specificity in its interaction with TFs (30, 31). Crystal structures of different DNA motifs have revealed that local structure of DNA helix is sequence dependent (2, 28, 32). Various studies have highlighted that fine structural features of DNA such as helical twist, groove shape, slide, roll etc. to be sequence dependent which appear to be determinants for specificity in protein–DNA recognition (7, 33, 34). Role of water is also very important in DNA–protein interactions as water is critical for B-form of DNA (6). This sequence dependent variability in the DNA geometry leads to localized variations in the depth and width of the major and minor groove of the DNA helix and can have subtle effect in imparting specificity in DNA–protein interactions which in turn may regulate the gene expression (2, 3).

An important question in chemical biology and molecular medicine is the designing of synthetic molecules that can sequence specifically bind in the genome (16, 35). Such molecules are designed to regulate biological processes such as transcription to control expression of aberrant gene in a diseased state like cancer (36, 37). However, a major limitation in the creation of such DNA binding small molecules is the lack of knowledge of all parameters of their DNA sequence-recognition. Obvious solution is to design longer molecules which will increase specificity. However, this has its own problem as designing a stiff longer molecule is a challenge in itself. Long molecule drugs also pose delivery problem. However, this problem can be easily overcome by using nanoparticles as delivery vehicle. This is why many synthetically designed drugs have undesired effects on the cells. Currently, there is no tool available which they can use to know the off target sites of the drug. Onco-Regulon fills this lacuna by allowing experimental biologists to design experiments with a better control as they will be in a position to check if the drug binds to non-specific targets as predicted by closest sequence finder programme. We believe that our programme can be a stepping stone in designing of sequence specific drugs by computational biologists as well as their testing by experimental biologists. This will in turn help elucidate the molecular codes of DNA–drug interactions.
